# Nutrient Deprivation Affects *Salmonella* Invasion and Its Interaction with the Gastrointestinal Microbiota

**DOI:** 10.1371/journal.pone.0159676

**Published:** 2016-07-20

**Authors:** Sophie Yurist-Doutsch, Marie-Claire Arrieta, Audrey Tupin, Yanet Valdez, L. Caetano M. Antunes, Ryan Yen, B. Brett Finlay

**Affiliations:** 1 Michael Smith Laboratories, University of British Columbia, Vancouver, BC, V6T 1Z4, Canada; 2 Department of Microbiology & Immunology, University of British Columbia, Vancouver, BC, V6T 1Z3, Canada; 3 Instituto Nacional de Ciência e Tecnologia de Inovação em Doenças Negligenciadas, Centro de Desenvolvimento Tecnológico em Saúde, Fundação Oswaldo Cruz, Rio de Janeiro, RJ, 21040–900, Brazil; 4 Escola Nacional de Saúde Pública Sergio Arouca, Fundação Oswaldo Cruz, Rio de Janeiro, RJ, 22780–194, Brazil; 5 Department of Biochemistry and Molecular Biology, University of British Columbia, Vancouver, BC, V6T 1Z4, Canada; New York State Dept. Health, UNITED STATES

## Abstract

*Salmonella enterica* serovar Typhimurium (*S*. Typhimurium) is a foodborne enteric pathogen and a major cause of gastroenteritis in humans. It is known that molecules derived from the human fecal microbiota downregulate *S*. Typhimurium virulence gene expression and induce a starvation-like response. In this study, *S*. Typhimurium was cultured in minimal media to mimic starvation conditions such as that experienced by *S*. Typhimurium in the human intestinal tract, and the pathogen’s virulence *in vitro* and *in vivo* was measured. *S*. Typhimurium cultured in minimal media displayed a reduced ability to invade human epithelial cells in a manner that was at least partially independent of the Salmonella Pathogenicity Island 1 (SPI-1) type III secretion system. Nutrient deprivation did not, however, alter the ability of *S*. Typhimurium to replicate and survive inside epithelial cells. In a murine model of *S*. Typhimurium-induced gastroenteritis, prior cultivation in minimal media did not alter the pathogen’s ability to colonize mice, nor did it affect levels of gastrointestinal inflammation. Upon examining the post-infection fecal gastrointestinal microbiota, we found that specifically in the 129Sv/ImJ murine strain *S*. Typhimurium cultured in minimal media induced differential microbiota compositional shifts compared to that of *S*. Typhimurium cultured in rich media. Together these findings demonstrate that *S*. Typhimurium remains a potent pathogen even in the face of nutritional deprivation, but nevertheless that nutrient deprivation encountered in this environment elicits significant changes in the bacterium genetic programme, as well as its capacity to alter host microbiota composition.

## Introduction

*Salmonella enterica* are a group of Gram-negative facultative intracellular pathogens responsible for a variety of human diseases, ranging from gastroenteritis to life-threatening typhoid fever [1][2]. It is presently estimated that 98.3 million people worldwide contract non-typhoidal *Salmonella* and 155,000 individuals die of the disease each year [3]. Exposure to non-typhoidal *Salmonella* strains is generally through oral ingestion, either of meat from animals colonised by the pathogen, or by fecal contamination [3]. *Salmonella* epidemics linked to contaminated fruits and vegetables have been shown to result from poor sanitation standards used by infected food handlers, as well as due to manure fertilization of crop fields [4][5].

*Salmonella enterica* serovar Typhimurium (*S*. Typhimurium), a leading cause of gastroenteritis in humans, relies on two distinct groups of genes for successful colonization of the host. Salmonella Pathogenicity Island 1 (SPI-1) encodes a type III secretion system and the secreted effectors required for host invasion through M-cells in Peyer’s patches along the small intestinal tract [6]. Proliferation inside host cells, on the other hand, requires Salmonella Pathogenicity Island 2 (SPI-2), which encodes a second type III secretion system and its subsequently secreted effectors [7] [8]. The expression of both pathogenicity islands is regulated by nutrient availability, which likely signal to the pathogen to allow it to recognize its spatial location. For example, SPI-2 is strongly activated by magnesium and phosphate limitation, a state that mimics the intracellular environment where SPI-2 expression is most essential [9].

In the gastrointestinal (GI) tract, a complex interaction takes place between the invading pathogen, the host immune system, and the community of commensal and symbiotic microorganisms colonizing the host (the microbiota) [10][11]. Antibiotic modulation of the intestinal microbiota composition prior to *Salmonella* infection has been used to induce gastroenteritis-like symptoms in the mouse model [12][13], with specific microbiota alterations linked to increased susceptibility to pathogen colonization and intestinal inflammation [14][15]. Infection with *S*. Typhimurium, in turn, leads to significant alterations in the GI tract microbiota populations [16]. GI inflammation, a protective host immune response to pathogen infiltration, has been shown to provide *S*. Typhimurium with vital nutrients while depleting the GI microbiota and thus effectively promotes pathogen intestinal colonization [17][13][18][19][20][21]. Additionally, a diverse gut microbiota community has been shown to be important for promoting pathogen clearance post-infection [22].

It has been previously reported that culturing *S*. Typhimurium in the presence of human fecal extract, rich in metabolites produced by both the host and the GI microbiota, results in drastic changes to the pathogen’s gene expression profile[23]. A downregulation of SPI-1 expression, alongside upregulation of amino acid biosynthetic pathways, especially the leucine biosynthesis operon, was observed in *S*. Typhimurium cultured in the presence of fecal extract [23]. However, how fecal metabolites induce this starvation-like response in *S*. Typhimurium, and what the functional effects of these gene expression changes are during infection, remains unclear.

In this present study, we used HeLa epithelial cells and the murine model of gastroenteritis to examine how nutrient deprivation affects *S*. Typhimurium virulence *in vitro* and *in vivo*. Additionally, we determined how nutrient deprivation affected the pathogen’s interactions with the host and the GI microbiota.

## Materials and Methods

### Bacterial strains and growth

*Salmonella enterica* serovar Typhimurium strains used in this study were SL1344 wild type (SL1344)[24], SL1344 Δ*invA* (Δ*invA*) [25] and SL1344 Δ*ssaR* (Δ*ssaR*)[26]. All strains were cultured in Luria-Bertani (LB) medium or Morpholinepropanesulfonic acid (MOPS) minimal medium [27] with glucose as the carbon source, supplemented with 5 μg/ml histidine (Sigma-Aldrich, St Louis, MO, USA).

### Quantitative real time PCR

Bacterial RNA was isolated using the RNeasy Mini Kit (Qiagen, Hilden, Germany). Contaminating DNA was removed with a DNAFree kit (Ambion, Austin, TX). cDNA was prepared using the QuantiTect Reverse Transcription Kit (Qiagen). Primers used in this study, *hilA* (F-5’-3’:ACACCTGCAGGATAATCCAA, R-5’-3’:ATTTTCGTGCCAGTTCATGT) *ssrA* (F-5’-3’:TAAGAGCCACCTGCCATTAG, R-5’-3’:TGAAAACGGCAATAAGTGGT) *leuA* (F-5’-3’:ATCTCCGTTCATACCCACGA, R-5’-3’:ACGTTCTCCGATACCGTTCA) were tested for qPCR efficiency and used at a final concentration of 400nM. PCR amplification was performed in an Applied Biosystems 7500 system (Applied Biosystems, Foster City, CA, USA) with the QuantiTect SYBR Green PCR Kit (Qiagen). Relative quantification was calculated by the ΔΔCt method where the glyceraldehyde-3-phosphate dehydrogenase (*gapA*)[28] was used as the house-keeping gene.

### Tissue culture

HeLa epithelial cells were purchased from the American Type Culture Collection (Manassas, USA) and grown in Dulbecco's Modified Eagle Medium (DMEM; HyClone, Waltham, USA) supplemented with 10% fetal bovine serum (FBS; HyClone), 1% non-essential amino acids (Gibco, Carlsbad, USA) and 1% GlutaMAX (Gibco). All experiments were performed in 24-well plates seeded with 5X10^4^ cells per well approximately 24 hours before the onset of infection. *S*. Typhimurium was cultured in LB or MOPS media to mid-logarithmic stage, centrifuged, resuspended in phosphate buffered saline (PBS) and diluted in tissue culture media. The multiplicity of infection was 10 for the SL1344 and Δ*ssaR* strains. The Δ*hilA* strain was used at multiplicity of infection 100. Cells were infected for 10 minutes at 37°C, 5% CO_2_ after which time they were washed with PBS and layered with fresh medium. Following a 20 minute incubation, the medium was substituted for one supplemented with 50 μg/ml of gentamycin (Sigma-Aldrich, St Louis, MO, USA). After 1.5 hours, the medium was changed again to one containing 10 μg/ml gentamycin. Cells were lysed in 250 μL of 1% Triton X-100 (BDH, Yorkshire, UK), 0.1% sodium dodecyl sulphate (Sigma-Aldrich, St Louis, MO, USA) at 2 hours or 4 hours post infection. At each time point, serial dilutions were plated on LB plates containing 100 μg/mL of streptomycin (Sigma-Aldrich) for bacterial enumeration. The replication index was calculated by dividing the CFU at 4 hours by the average CFU at 2 hours post infection.

### Animal studies

All mouse experiments were approved by, and performed in strict accordance with the guidelines of the University of British Columbia Animal Care Committee and the Canadian Council on the Use of Laboratory Animals. All animal experiments were performed on female, age-matched (6–8 weeks old) mice. C57BL/6 mice were purchased from Jackson Laboratory (Bar Harbor, ME, USA). 129Sv/ImJ mice were bred in in-house at the University of British Columbia. Mice were treated with 450 mg/L of streptomycin (Sigma-Aldrich, St Louis, MO, USA) in drinking water for 2 days prior to infection, after which they were placed on regular drinking water. Mice were orally gavaged with 5*10^^4^ CFU mid-logarithmic state *S*. Typhimurium, in phosphate buffered saline. Mice were euthanized 3 days post infection by anaesthesia with isoflurane followed by CO_2_ asphyxiation and organs were manually dissected for further analysis. All animals used in this study displayed expected and reasonable level of morbidity and survived to the experimental end point.

### Murine sample collection and assessment of *S*. Typhimurium colonization

Ceca, colons and spleens were collected in 1 mL of sterile phosphate-buffered saline (PBS) and homogenized in a FastPrep Homogenizer (MP Biochemicals). Serial dilutions were plated in LB agar plates containing 100 μg/mL of streptomycin (Sigma-Aldrich, St Louis, MO, USA) for *S*. Typhimurium CFU enumeration. The cecal homogenate was centrifuged and supernatants were separated to be used in a Cytokine Bead Array. Fecal pellets were collected for 16S rDNA sequencing.

### Cytokine bead array

A cytometric bead assay (CBA) for mouse inflammation (BD Biosciences, Franklin lakes, NJ, USA) was used to analyse mouse inflammatory cytokine production (IL-6, IL-10, MCP-1, IFN-*γ*, TNF, IL-12p70) according to manufacturer’s instructions.

### Statistical analysis

Analysis was performed using Prism7 (GraphPad, CA, USA). qPCR and tissue culture p-value was calculated using the students T-test. For S. Typhimurium colonization in mice and cecal cytokine levesl, p-value was calculated using the Mann-Whitney U test. Principal component analysis of the microbiota was statistically compared by Permanova. The Shannon diversity index was calculated using Phyloseq and statistically confirmed by Mann-Whitney (GraphPad Prism software, version 5c, CA). Differentially abundant taxa were statistically compared using DEseq2 (50) under default settings. The test statistics’ p-values were adjusted for multiple testing using the procedure of Benjamini and Hochberg (false discovery rate threshold = 5%).

### 16S Microbial community analysis

DNA was extracted from 1–2 pellets of mouse stool. Samples were mechanically lysed using MO BIO dry bead tubes (MO BIO Laboratories) and the FastPrep homogenizer (FastPrep Instrument, MP Biochemicals) before DNA extraction with the Qiagen DNA Stool Mini Kit.

All samples were amplified by PCR in triplicate using barcoded primer pairs flanking the V3 region of the 16S gene as previously described [29]. Each 50 μl of PCR contained 22 μl of water, 25 μl of TopTaq Master Mix, 0.5 μl of each forward and reverse barcoded primer, and 2 ml of template DNA. The PCR program consisted of an initial DNA denaturation step at 95°C for (5 min), 25 cycles of DNA denaturation at 95°C (1min), an annealing step at 50°C (1min), an elongation step at 72°C (1 min), and a final elongation step at 72°C (7 min). Controls without template DNA were included to ensure that no contamination occurred. Amplicons were run on a 2% agarose gel to ensure adequate amplification. Amplicons displaying bands at ~160 bp were purified using the illustra GFX PCR DNA Purification kit. Purified samples were diluted 1:50 and quantified using PicoGreen (Invitrogen) in the TECAN M200 (excitation at 480 nm and emission at 520 nm).

Pooled PCR amplicons were diluted to 20 ng/ml and sequenced at the V3 hypervariable region using Hi-Seq 2000 bidirectional Illumina sequencing and Cluster Kit v4 (Macrogen Inc.). Library preparation was done using TruSeq DNA Sample Prep v2 Kit (Illumina) with 100 ng of DNA sample and QC library by Bioanalyzer DNA 1000 Chip (Agilent).

Sequences were preprocessed, denoised, and quality filtered by size using Mothur [30]. Representative sequences were clustered into OTUs using CrunchClust [31] and classified against the Greengenes Database [32] according to 97% similarity. Any OTUs present less than five times among all samples were removed from the analysis.

Fecal microbial diversity, principal components analysis (PCA) and the relative abundance of bacterial taxa were assessed using Phyloseq [33] along with additional R-based computational tools in R-studio (R-Studio, Boston, MA).

## Results

### *S*. Typhimurium cultured in minimal media displays a gene expression profile similar to fecal extract supplemented medium

Previous work from our laboratory has demonstrated that *S*. Typhimurium starvation genes are activated in the presence of human fecal extract [23]. Fecal matter is incredibly complex in terms of molecular composition [34] and its exact composition can vary based on the donor and the timing of collection. In an effort to dissect the role of starvation in *S*. Typhimurium pathogenesis we chose to grow the bacteria in Morpholinepropanesulfonic acid (MOPS) minimal media, in comparison with a control, nutrient rich media (Luria-Bertani (LB)). Culturing *S*. Typhimurium in MOPS minimal media has been reported to induce an upregulation in the expression of amino acid biosynthesis pathway genes and repress the expression of the SPI-1 and SPI-2 operons [35], a gene expression pattern similar to what has been observed in the presence of fecal extract [23].

**Fig 1:**
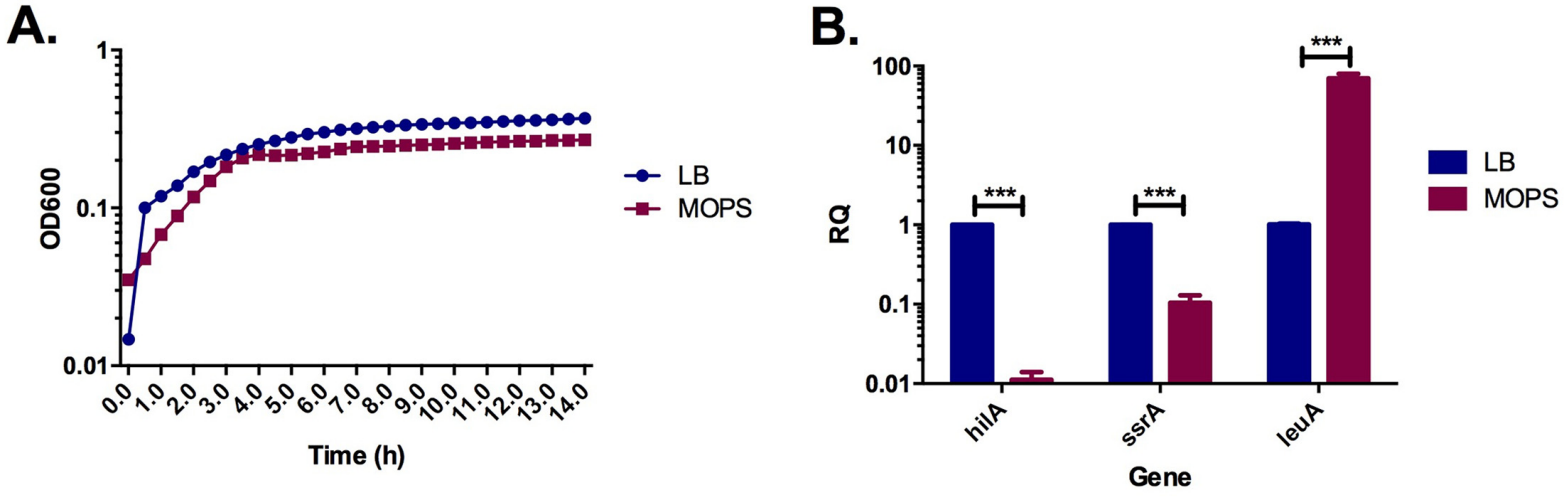
*S*. Typhimurium cultured in MOPS minimal media displays similar growth and an altered gene expression profile when compared to culture in rich media. A. Growth over time was monitored by measuring optical density at 600nm. Results represent the average of three independent measurements (n = 3). B. Logarithmic state bacteria were collected for RNA extraction and the relative quantification of *hilA*, *ssrA* and *leuA* in LB (blue) and MOPS (red). Results represent the average of three independent measurements (n = 3). ***, p<0.001.

We first measured the viability and growth rate of *S*. Typhimurium in MOPS minimal media compared to LB media. As reflected in Fig 1a, with an adjustment of the initial culture density, *S*. Typhimurium cultured in minimal or rich media reaches late logarithmic state at roughly 3 hours post inoculation and no major differences could be observed between the two growth conditions.

To confirm previous findings that *S*. Typhimurium cultured in MOPS minimal media displays upregulation of amino acid biosynthesis genes and downregulation of virulence factors [35], quantitative real time PCR was used to estimate the relative expression of 3 representative genes: 1) *hilA*–the main regulator of the SPI-1 secretion system [36] 2) *ssrA*–one of the main regulators of SPI-2 [7][37] and 3) *leuA*–the first gene of the leucine biosynthesis operon [38]. As expected, growth in minimal media led to a ~70 fold upregulation of *leuA*, while the virulence regulators, *ssrA* and *hilA*, were strongly repressed by ~10 and ~100 fold respectively in the nutrient deprived conditions respectively (Fig 1b).

### *S*. Typhimurium cultured in minimal media displays reduced invasion in human epithelial cells

We next aimed to determine the effect of nutrient deprivation on *S*. Typhimurium *in vitro*. Upon ingestion by the host, *S*. Typhimurium infection cycle begins with invasion of epithelial cells along the host GI tract [6]. To examine this cycle, invasion and intracellular replication of LB and MOPS cultured *S*. Typhimurium was measured in a human epithelial cell line (HeLa).

**Fig 2:**
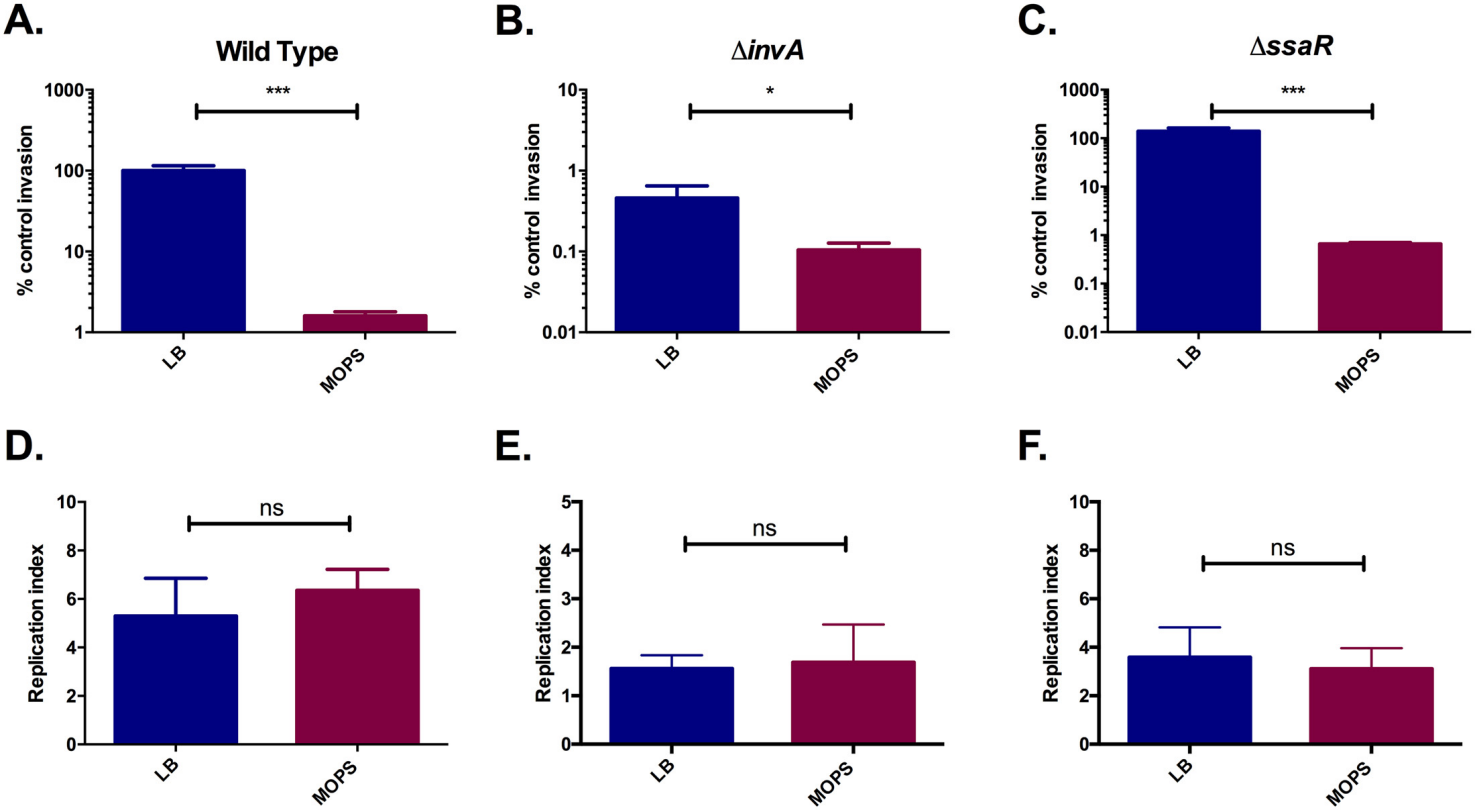
Growth in minimal media alters the ability of *S*. Typhimurium to invade, but not replicate in epithelial cells. HeLa cells were grown in Dulbecco’s modified Eagle’s medium supplemented with 10% fetal bovine serum, 1% nonessential amino acids, and 1% GlutaMAX. Wildtype, Δ*invA* and Δ*ssaR S*. Typhimurium were cultured to mid-logarithmic phase in LB (blue) or MOPS minimal media (red). HeLa cells invasion was measured at 2 hours post infection and is represented as a precent of Wild Type *S*. Typhimurium cultured in LB (control). The replication index was calculated by dividing the CFU/well at 4 hours post infection with the CFU/well at 2 hours post infection. A. Wildtype, invasion. B. Δ*invA*, invasion. C. Δ*ssaR*, invasion. D. Wildtype, replication. B. Δ*invA*, replication. C. Δ*ssaR*, replication. Results represent the average of four independent measurements (n = 4). *, p<0.05. ***, p<0.001. ns, not significantly different.

*S*. Typhimurium cultured in MOPS media was able to invade HeLa cells at ~100 fold less than the invasion level of LB cultured bacteria (Fig 2a). This is consistent with previous studies demonstrating that SPI-1 repression, specifically by fecal conditions, results in a reduced ability to invade epithelial cells [39][23].

To test whether nutrient deprivation itself contributes to the drastic reduction in HeLa cell invasion or if this is simply the result of SPI-1 downregulation, the epithelial cell invasion assay was repeated with an *invA* deletion mutant. The *invA* deletion mutant lacks a key component of the SPI-1 secretion system that was previously demonstrated to be impaired in epithelial cell invasion[40] [41] [42]. As previously reported, the *invA* mutant was able to invade HeLa cells at a fraction of the rate of the wild type strain (Fig 2b). Nevertheless, culturing the SPI-1 mutant in MOPS media resulted in a significant decrease in HeLa cell invasion in comparison to the LB control (Fig 2b), albeit to a much lesser extent than the one observed with the wild type strain. These data suggest that starvation conditions inhibit the ability of *S*. Typhimurium to invade epithelial cells by mechanisms that are at least in part independent of SPI-1 repression. The invasion ability of a SPI-2 mutant (Δ*ssaR* [43]) was also measured. When cultured in MOPS media Δ*ssaR S*. Typhimurium displayed an invasion defect similar to the one observed for the wild type *S*. Typhimurium strain (Fig 2C), suggesting that starvation conditions inhibit the invasion ability of *S*. Typhimurim independently of SPI-2.

We next aimed to determine whether the ability of *S*. Typhimurium to survive inside cells was affected by prior culture in MOPS minimal media. To measure intracellular replication, we divided the number of *S*. Typhimurium colony forming units (CFU) inside HeLa cells at 4 hours post-infection by that at 2 hours post-infection. A short time point was selected as we hypothesised that if prior culture media affects intracellular survival, it will be at a time close to the initial invasion point. No significant differences were detected in the replication index of wild type, Δ*invA* or Δ*ssaR S*. Typhimurium in MOPS-cultured compared with LB-cultured bacteria (Fig 2D–2F). As such, the down regulation of SPI-2 in nutrient limiting conditions do not appear to affect the ability of *S*. Typhimurium to survive or replicate in epithelial cells.

### Minimal media cultivation does not affect *S*. Typhimurium colonization or colitis in a mouse model of murine gastroenteritis

Since *S*. Typhimurium cultured in minimal media displays reduced invasion in cultured epithelial cells, we next set out to test whether these culture conditions affected the virulence of *S*. Typhimurium *in vivo*.

**Fig 3:**
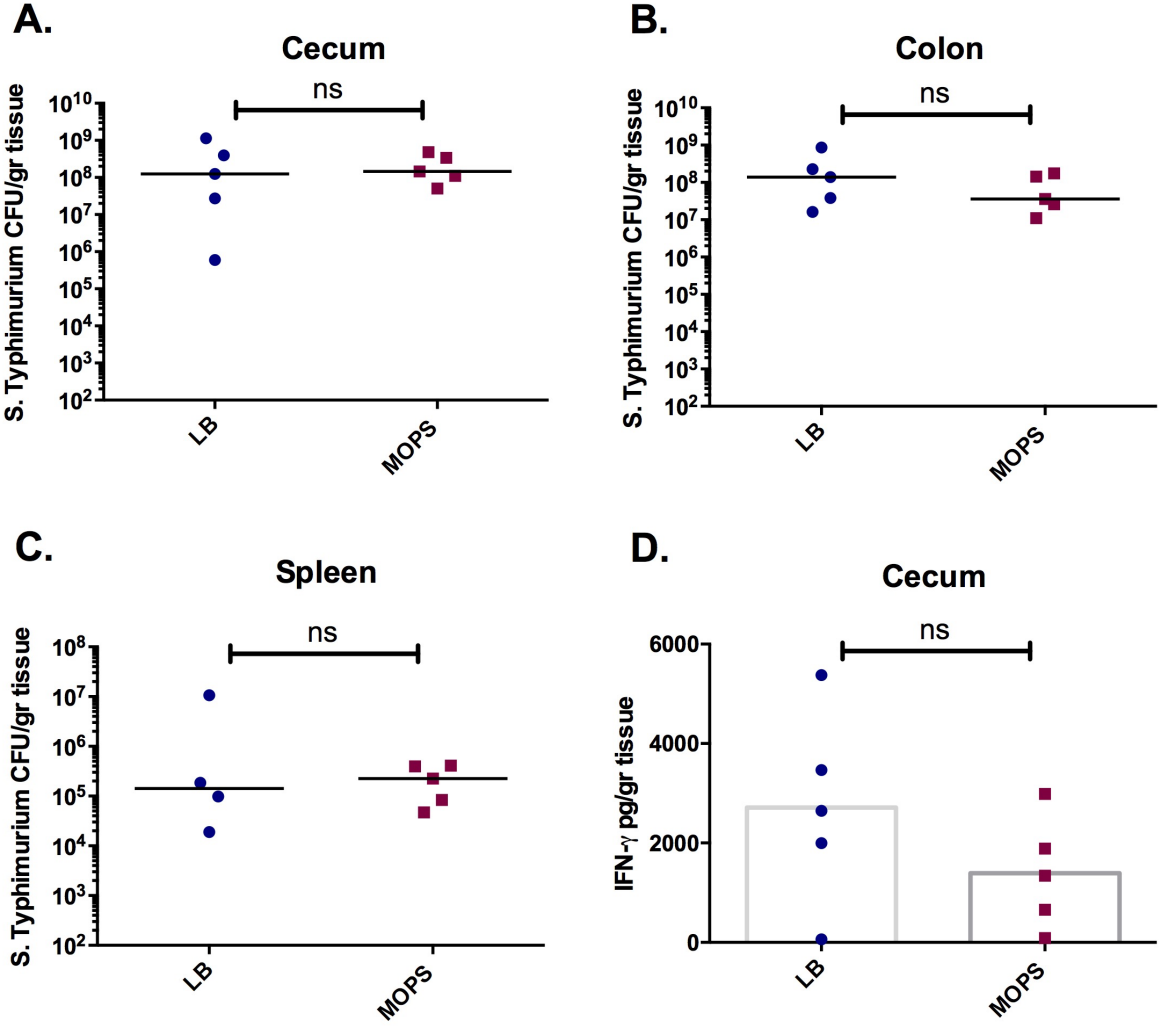
Growth in minimal media does not affect the ability of *S*. Typhimurium to colonize and induce inflammation in the gut of C57BL/6 mice. Streptomycin treated mice were infected with wild type *S*. Typhimurium cultured to mid-logarithmic phase in LB (blue) or MOPS minimal media (red). *S*. Typhimurium colonization of the cecum (A), colon (B) and spleen (C). D. Levels of IFN-γ in the cecum. Experiments were performed 3 times with a minimum of five mice per test group. ns, not significantly different.

C57BL/6 mice, pre-treated with streptomycin to induce susceptibility to gastroenteritis [13], were infected with late-logarithmic phase wild type *S*. Typhimurium cultured in LB or MOPS media. Mice were sacrificed 3 days post-infection and the cecum, spleen and colon were examined for *S*. Typhimurium colonization levels. No significant differences in pathogen colonization were observed in the GI tract (Fig 3a and 3b) or spleen (Fig 3c) of mice infected with LB or MOPS cultured bacteria. Furthermore, levels of GI inflammation, as reflected by the levels of IFN-γ in the cecal homogenate, were not significantly altered between mice infected with MOPS- or LB-cultured bacteria (Fig 3d). Levels of TNF-α, IL-6, IL-10, IL12-p70 and MCP-1 in the cecal homogenate were also unchanged between the test conditions (data not shown).

**Fig 4:**
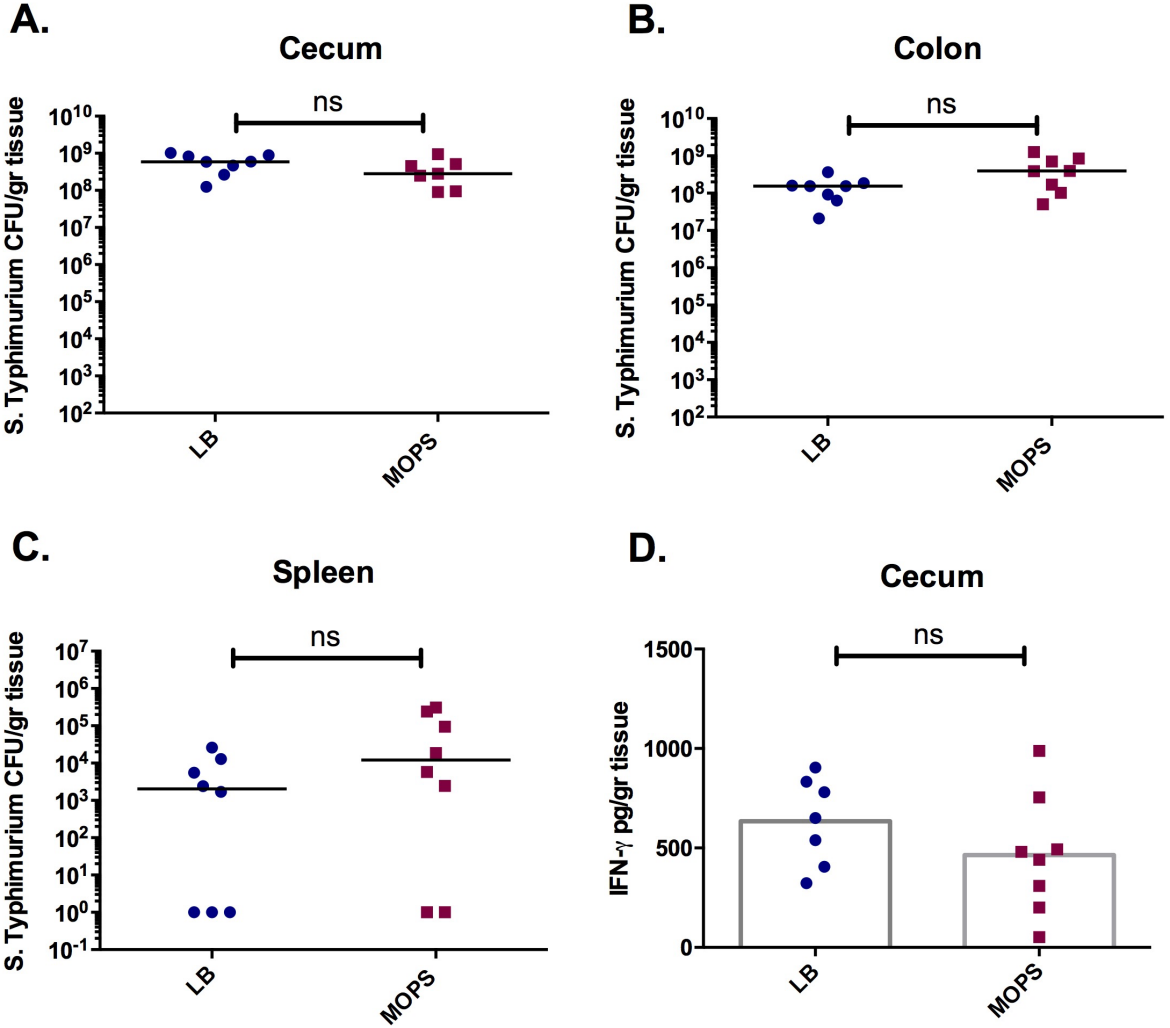
Growth in minimal media does not affect the ability of *S*. Typhimurium to colonize and induce inflammation in the gut of 129Sv/ImJ mice. Streptomycin treated mice were infected with wild type *S*. Typhimurium cultured to mid-logarithmic phase in LB (blue) or MOPS minimal media (red). *S*. Typhimurium colonization of the cecum (A), colon (B) and spleen (C). D. Levels of IFN-γ in the cecum. Experiments were performed 3 times with a minimum of five mice per test group. ns, not significantly different.

The 129Sv/ImJ murine strain is naturally more resistant to *S*. Typhimurium colonization than C57BL/6 mice, which is largely attributed to presence of the Nramp (Natural resistance-associated macrophage protein 1) gene in 129Sv/ImJ mice [44]. When pre-treatment with streptomycin is used to induce susceptibility to gastroenteritis, similarly to the C57BL/6 strain, 129Sv/ImJ mice display peak gastric colonization and colitis at 3 days post infection [45][44]. However, in 129Sv/ImJ mice systemic pathogen colonization levels are low and the mice can survive the infection [44]. The progression of disease in 129Sv/ImJ mice is thus more similar to *Salmonella*-induced gastroenteritis in humans than the acute terminal illness observed in C57BL/6 mice. We next tested whether starvation conditions had an effect on *S*. Typhimurium virulence in 129Sv/ImJ mice. Streptomycin pre-treated mice were infected with late-logarithmic stage *S*. Typhimurium cultured in rich or minimal media. Similar to our results in C57BL/6 mice, at 3 days post infection no differences were detected in *S*. Typhimurium burden in the cecum (Fig 4a), colon (Fig 4b) or spleen (Fig 4c) of mice infected with MOPS- or LB-cultured *S*. Typhimurium. Additionally, levels of cecal IFN-γ (Fig 4d), and other pro-inflammatory cytokines (TNF-α, IL-6, IL-10, IL12-p70 and MCP-1; data not shown) were similar between the test groups indicating no difference to *S*. Typhimurium-induced inflammation.

### Culture media alters *S*. Typhimurium induced microbiota compositional shifts but not levels of colonization in a mouse strain-specific manner

**Fig 5:**
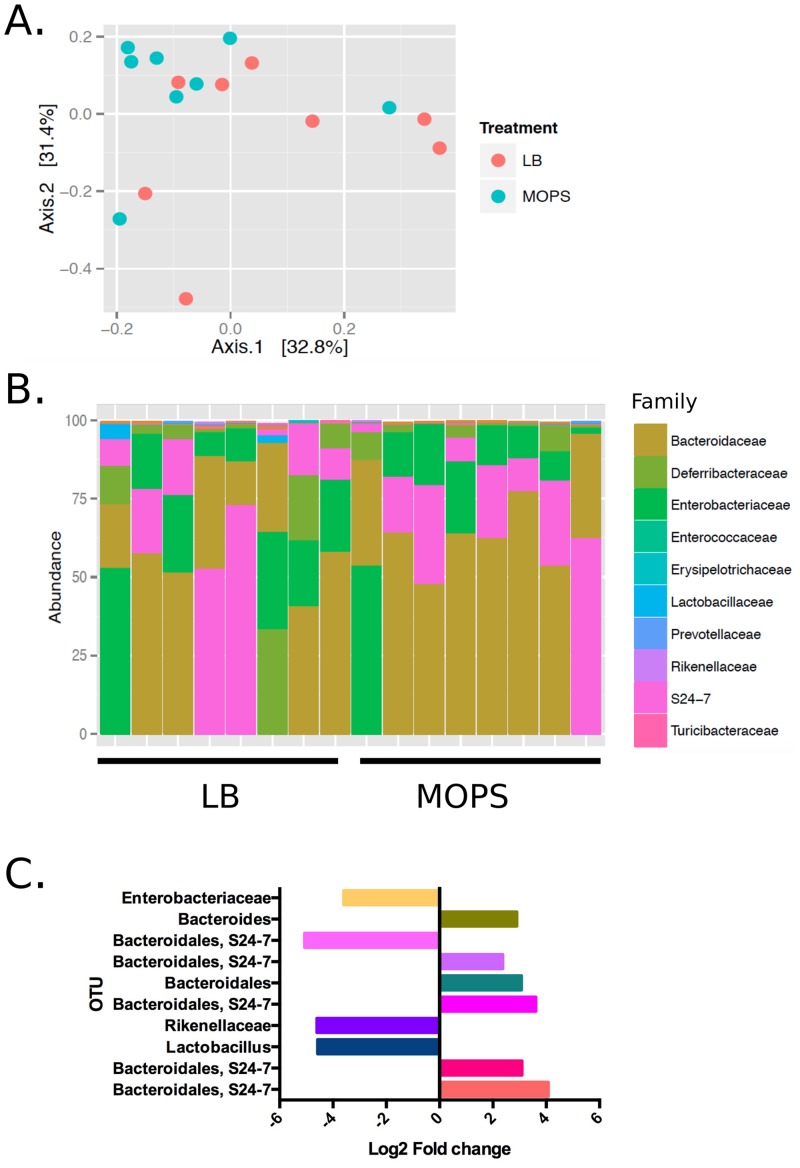
The gastrointestinal microbiota composition and diversity of 129Sv/ImJ mice after infection with LB or MOPS cultured *S*. Typhimurium. A. Multivariate analysis by PCA of the fecal microbiota of 129Sv/Imj mice infected with SL1344 cultured in LB or MOPS. B. Relative abundance of bacterial families within the top 100 OTUs in the fecal microbiota. Colors of rectangles correspond to the bacterial families in the legend. C. Log fold change of OTUs significantly (FDR<0.05) different between the LB and MOPS test groups.

We next characterised how *S*. Typhimurium that had been pre-cultured in minimal or rich media differentially affected GI microbiota populations by sequencing the 16S rRNA gene in the feces of *S*. Typhimurium-infected mice. In 129Sv/ImJ mice, as reflected in a PCA plot (Fig 5a), no significant differences could be measured in the beta diversity. Additionally, the culture media used to grow the pathogen did not affect family distribution in the GI microbiota of these mice (Fig 5b). However, there were significant differential taxa at the OTU-level (Deseq, Wald test corrected with FDR); Specifically, 10 OTUs were different between the LB and MOPS infection conditions (Fig 5c). While four OTUs belonging to the Bacteroidales S24-7 group were elevated in the MOPS test group, one OTU of this group was actually higher in the LB infection condition. *Bacteroides* and *Bacteroidales* OTUs were also elevated in the MOPS infection group while *Lactobacillus*, Rikenellaceae, and Enterobacteriaceae OTUs were are all reduced in the fecal microbiota of mice infected with SL1344 cultured in minimal media.

**Fig 6:**
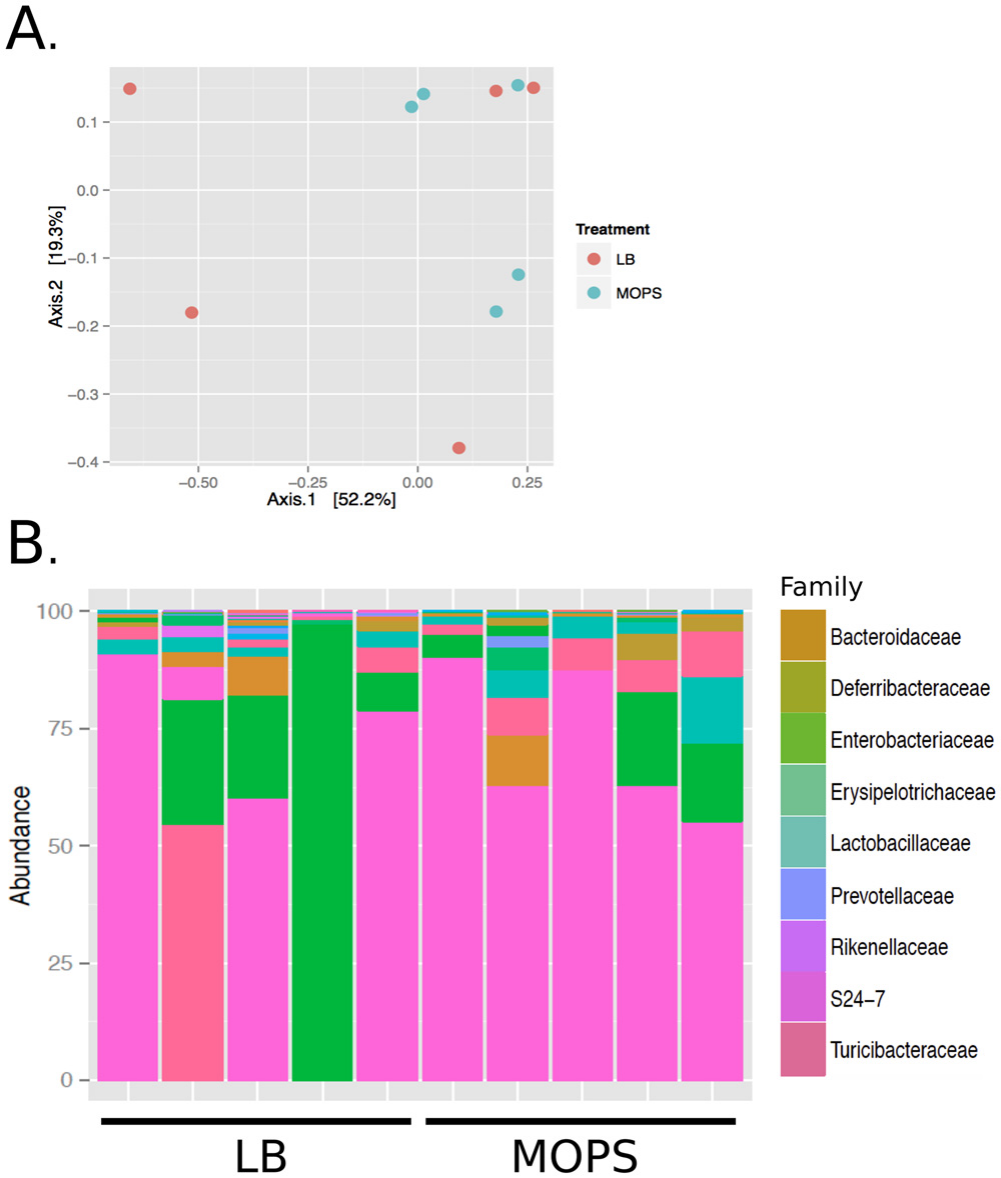
The gastrointestinal microbiota composition and diversity of C57BL/6 mice after infection with LB or MOPS cultured SL1344. A. Multivariate analysis by PCA of the fecal microbiota of C57BL/6 mice infected with SL1344 cultured in LB or MOPS. B. Relative abundance of bacterial families within the top 100 OTUs in the fecal microbiota. Colors of rectangles correspond to the bacterial families in the legend.

We performed a similar 16S rRNA analysis of the microbiota populations of C57BL/6 mice that had been infected with LB- or MOPS- cultured *S*. Typhimurium. Like the 129Sv/ImJ strain, no significant differences in beta diversity (Fig 6a) or family distribution (Fig 6b) could be measured. Furthermore, none of the 488 OTUs detected in the sequencing reaction were significantly different between mice that had been infected with MOPS- or LB- cultured *S*. Typhimurium.

## Discussion

Non-typhoidal salmonellosis remains a global health problem [3]. Due to a broad range of potential hosts, sources of *Salmonella* contamination can vary from meat of infected animals to food and water contaminated with the feces from human or animal carriers of the disease. Due to its exposure to diverse environmental conditions throughout its lifecycle, it is reasonable to predict that S. Typhimurium may experience temporary or long-term nutrient deprivation at some point.

In our study, we aimed to elucidate the role of nutrient deprivation during infection. We found that in epithelial cells, cultivation in minimal media reduces the ability of *S*. Typhimurium to invade host cells in a SPI-1 and SPI-2 independent manner. This finding indicates that nutrient limitation itself can dampen virulence. However, during an *in vivo* model of *S*. Typhimurium gastroenteritis, prior cultivation of *S*. Typhimurium in minimal media did not significantly affect pathogen burden in the GI tract, nor did it affect levels of intestinal inflammation, or systemic pathogen transfer.

Nutrient limitation can have a complex effect on *S*. Typhimurium. Selective pressure caused by starvation can lead to genetic variability in the pathogen community, and may result in chromosomal gene duplication [46]. Furthermore, in enterobacteria such as *E*. *coli* O157/H7, starvation has been shown to increase acid tolerance [34], which is expected to contribute to *in vivo* virulence. Our data, however, indicates that short-term starvation prior to infection, does not affect the ability of *S*. Typhimurium to cause gastrointestinal and systemic disease. It would appear that contact with the GI environment effectively overwrites the starvation experienced by the pathogen prior to infection of the host.

We did find, however, that prior culture of *S*. Typhimurium in minimal media resulted in an altered post-infection GI microbiota composition, compared with *S*. Typhimurium cultured in rich media. Infection with *S*. Typhimurium grown in nutrient limiting conditions resulted in an increase of *Bacteroidetes* species and a decrease of Enterobacteriaceae microbiota members in the fecal microbiota when compared to a rich media grown pathogen infection. In human populations, non-typhoidal *Salmonella* infection has been shown to lead to a massive decrease in *Bacteroidetes* populations [16], and as thus our results suggest that infection with *S*. Typhimurium grown in rich media better models human disease.

The underlining mechanisms of GI microbiota compositional shifts selectively induced by minimal versus rich media cultured pathogen remain unclear. It is possible that while classical indicators of colitis, such as levels of pro-inflammatory cytokines in the gut, do not change between the infection conditions, the host response to infection still varies and thereby differentially affects the GI microbiota. Alternatively, metabolic changes to *S*. Typhimurium after cultivation in different media may alter microbiota composition through direct bacteria-bacteria interactions. Minimal versus rich media cultivation has been previously shown to change the metabolic profile of any given organism [47].

Minimal media cultivation, as a model of infection resulting from fecal contamination or water borne pathogen, reveals *S*. Typhimurium to be an extremely potent pathogen that can overcome nutrient deprivation and virulence gene repression to successfully colonize the host. It further reveals that the interaction between the pathogen and the GI microbiota may depend on the metabolic state of the invading pathogen.
